# PSSM-based prediction of DNA binding sites in proteins

**DOI:** 10.1186/1471-2105-6-33

**Published:** 2005-02-19

**Authors:** Shandar Ahmad, Akinori Sarai

**Affiliations:** 1 Department of Bioinformatics and Bioscience, Kyushu Institute of Technology, Iizuka 820 8502, Fukuoka, Japan; 2 Department of Biosciences, Jamia Millia Islamia University, New Delhi-110025, India

## Abstract

**Background:**

Detection of DNA-binding sites in proteins is of enormous interest for technologies targeting gene regulation and manipulation. We have previously shown that a residue and its sequence neighbor information can be used to predict DNA-binding candidates in a protein sequence. This sequence-based prediction method is applicable even if no sequence homology with a previously known DNA-binding protein is observed. Here we implement a neural network based algorithm to utilize evolutionary information of amino acid sequences in terms of their position specific scoring matrices (PSSMs) for a better prediction of DNA-binding sites.

**Results:**

An average of sensitivity and specificity using PSSMs is up to 8.7% better than the prediction with sequence information only. Much smaller data sets could be used to generate PSSM with minimal loss of prediction accuracy.

**Conclusion:**

One problem in using PSSM-derived prediction is obtaining lengthy and time-consuming alignments against large sequence databases. In order to speed up the process of generating PSSMs, we tried to use different reference data sets (sequence space) against which a target protein is scanned for PSI-BLAST iterations. We find that a very small set of proteins can actually be used as such a reference data without losing much of the prediction value. This makes the process of generating PSSMs very rapid and even amenable to be used at a genome level. A web server has been developed to provide these predictions of DNA-binding sites for any new protein from its amino acid sequence.

**Availability:**

Online predictions based on this method are available at

## Background

There has been a growing interest in the prediction of DNA-binding sites in proteins which play crucial roles in gene regulation [1-4]. We have previously developed a method of predicting DNA-binding sites of proteins from the sequence information [5]. We reported development of a neural network and corresponding web server to predict amino acid residues which are likely to bind DNA. The only input to the neural network in this algorithm was the identity of the amino acid residue and its two sequence neighbors on C- and N- terminals. We also developed a method to identify DNA-binding proteins using electrical moments from structural information of proteins [6]. On the other hand, several investigators have reported that the use of evolutionary information in sequence-based predictions of secondary structure and solvent accessibility can improve the prediction capacity of a neural network [7-10].

Here we report the use of such evolutionary information in improving the prediction of DNA-binding sites of proteins. We note that one of the major problems in applying evolutionary information by way of position specific scoring matrices (PSSMs) for sequence based prediction is that such matrices are generated over large data sets and take a long time to complete. Thus large scale predictions remain inaccessible to moderately capable computers. This is a serious limitation in the portability of neural network based predictions using PSSMs [8]. In this work, we report that evolutionary profiles or PSSMs against much smaller representative reference data sets may be utilized to achieve almost the same levels of prediction as would be obtained from alignments with large sequence data sets representing entire available sequence space. We have used four different reference data sets of PSSMs for 62 representative protein sequences. These are (1) PDNA-RDN: a data set of protein sequences from all Protein-DNA complexes from the PDB, (2) PDNA-NR90: a non-redundant data set compiled from PDNA-RDN, (3) PDB-ALL: a data set of all amino acid sequences from PDB and (4) NCBI-NR: a non-redundant data set of all protein sequences taken from sequence and structure databases and compiled by NCBI (see Methods). We find that the net prediction (an average of sensitivity and specificity) of the best of these systems (using PIR sequence data as reference) improves to 67.1% from the value of 58.4% reported earlier for a sequence-only prediction. We also report that a small reference data set of 375 sequences (PDNA-NR90) can give a 64.6% net prediction – just 2.5% poorer than the best- while reducing the PSSM calculation time from more than two hours (against NCBI-NR) to just about one minute. A better compromise could be the use of PDNA-RDN data for which 65.2% net prediction #150; 1.9% less than the best- was obtained, while about 2 and a half minutes are taken to generate their PSSMs. It is also reported that the presence of redundancy is helpful in improving the prediction whereas presence of data not relevant for DNA-binding may in some cases reduce predictive performance.

## Results and discussion

Position Specific Iterative BLAST (PSI BLAST) is a strong measure of residue conservation in a given location. In the absence of any alignments, PSI BLAST simply returns a 20-dimensional vector representing probabilities of conservation against mutations to 20 different amino acids including itself. A matrix consisting of such vector representations for all residues in a given sequence is called Position Specific Scoring Matrix or PSSM. When a residue is conserved through cycles of PSI BLAST, it is likely to be due to a purpose i.e. biological function. It has been established by several authors cited in the introduction that the prediction of structural properties is significantly enhanced by the use of PSSMs compared to predictions based on unique representations of amino acid sequence and its environment. Protein structure universe is vast and a prediction of structural properties should span the entire range of this diversity. However, the question of predicting DNA-binding sites is much narrower and hence the significance of conservation of residues at specific locations may be limited to a subset of this protein space. Such reduction in the protein search space or the reference data sets against which PSSM-based predictions should be attempted is desired for a rapid prediction of binding sites as well as portability of prediction methods. Compact reference data size can not only answer these questions of speed and portability but also try to minimize noise in information contents and improve prediction quality.

Table 1 shows the results of DNA-binding site prediction using different sets of PSSMs as the neural network inputs. The best net prediction results were 67.1% which is 8.7% better than the predictions with sequence information only. These results were obtained for PSSMs against PIR sequence data. An even larger NCBI-NR data set showed a slightly smaller (66.7%) net prediction. The fact that NCBI-NR reference data sets produce somewhat worse results than PIR sequence suggests that the redundancy present in the PIR sequence data could be the factor responsible for giving better PSSMs than those of a non-redundant NCBI sequence data. Thus an overall redundancy in the data turns out to be helpful in improving the prediction of binding sites. The question is how rapidly the prediction ability will fall if we reduce the redundancy even further, replacing the larger data sets with smaller ones until a small representative data set is left. This question is partly answered by first using a sequence database of the entire protein data bank (PDB-ALL), which gives an accuracy of 64.7% (about 2.4% poorer than the best). Further reducing the data set to protein sequences from only the Protein-DNA complexes surprisingly increases the net prediction to 65.2%. We suggest that the increase in net prediction on the PDNA-RDN over the entire PDB is caused by the fact that PDNA-RDN contains all the data from PDB which is relevant for the DNA-binding. However, an additional data in the PDB-ALL represents conservation scores in regions not involved in DNA-binding and hence lead to a somewhat lower net prediction. Going further down from a redundant (PDNA-RDN) to a non-redundant (PDNA-NR90) sequence data of Protein-DNA complexes, we observe a 0.6% fall in net prediction- just about the same we observed from PIR to NCBI-NR. We attribute this fall in net prediction to the reduction in the redundancy in the sequence data sets, which is concluded to be useful in better prediction of DNA-binding sites.

In terms of CPU time, it may be noted that the time taken by 62 protein sequences used here is about one hour for the best (PIR) data sets. These times are prohibitively large for making predictions at a genomic scale or for providing rapid web services. A compromise could be obtained by using PDNA-RDN instead, which reduces the CPU time by a factor more than 8. The loss of net prediction for this compromise is about 1.9%, which is still 6.8% better than the predictions obtained from sequence information only. PSSMs against this data set for a typical protein of 500 residues can be generated in about 1 s, making it possible to run large scale predictions. A smaller size of reference data and high speed of PSSMs also make this method portable and light weight with a strong predictive ability.

Binary decision function of the neural network (see Methods) assigns a value of zero (not binding) or 1 (binding) based on a threshold on the real value output received at the output node. Most of the accuracy scores presented here have been obtained by using 0.5 as the cutoff (mid point of the transfer function range). By changing this threshold from 0.5 to higher and lower values, the balance between sensitivity and specificity can be adjusted. In our online prediction we also present the scores obtained for a ROC analysis of such adjustments (Figure 2). ROC for only one reference data set has been shown here as most other graphs show a similar behavior.

## Online predictions

We have provided online predictions based on the above method at our web site [16]. The raw probability scores, their annotations at different sensitivity thresholds, and a reference scale for expected sensitivity and specificity have been provided. In addition, results of sequence alignments obtained after PSI BLAST iterations against a reference data (PDNA-RDN) are also provided. This allows us to have a complete picture of similarity of a given sequence with known DNA-binding proteins and predictions based on neural network using alignment profiles in the form of PSSM. The only input to this neural network is the amino acid sequence of the protein. The web server will automatically generate PSSMs of the given sequence against a reference data and use them as the input to a neural network, trained for predictions of 62 DNA-binding proteins.

## Conclusion

A PSSM-based neural network method for predicting DNA-binding sites in proteins has been developed. PSSMs were developed against different data sets and it was observed that significant computer time can be saved by replacing the reference data sets with much smaller reference data sets without loss of much prediction ability. Redundant reference data sets show a better prediction than the non-redundant data sets. A web server was developed to provide prediction of DNA-binding sites based on this method. In addition, the web server provides BLAST alignments against a reference data set of known DNA-binding proteins.

## Methods

### Data sets

#### PDNA-62

This is the (non redundant) target data set of 62 DNA-binding proteins from Protein Data Bank (PDB) [11]. The same data set has been used in our related studies [5,12].

#### PDNA-RDN

This is a new data set, developed for this work. We have selected all Protein-DNA complexes from PDB and separated their chains. 1386 protein chains were obtained in this way. FASTA formatted sequences were subsequently formatted using *formatdb *program of the BLAST package [13].

#### PDNA-NR90

The data set (*PDNA-RDN*), obtained from the procedure mentioned above was filtered to remove redundancy at 90% sequence identity level by using sequence clustering program BLASTCLUST [13]. Resulting data set now contains 375 sequences which are formatted for use as a reference data set using *formatdb*. This data set is called *PDNA-NR90*.

#### Other data sets

*PDB-ALL *(47,189 sequences) is a data set of all protein sequences obtained from NCBI. *PIR *is the sequence data set (283,177 sequences) of Protein Information Resource at Georgetown University [14]. NCBI-NR is a non-redundant data set of all protein sequences compiled from GeneBank, PIR, SwissProt, PDB and other resources by NCBI [17].

### Generation of PSSMs

Target sequences are scanned against the reference data sets to compile a set of alignment profiles or position specific scoring matrices (PSSMs) using Position Specific Iterative BLAST (PSI BLAST) program [15]. Three cycles of PSI-BLAST were run for each protein and the scores were saved as profile matrices (PSSMs).

### Neural network

#### Neural network inputs

**Figure 1:**
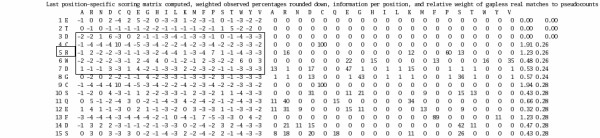
Rows of Position Specific Scoring Matrices selected for neural network input: Network inputs consist of the PSSM of the target residue and its two neighboring residues on C- and N-terminals. Each residue is thereby represented by a 20 dimensional vector with integer values. These values represent (logarithmic) effective frequencies of occurrence at respective positions in a multiple alignment. Neural network input layer is therefore made of 20 × 5 = 100 units. Two units in the only hidden layer and one unit in the output layer add up to a total of 202 neural units to be trained in the fully connected neural network.

**Figure 2:**
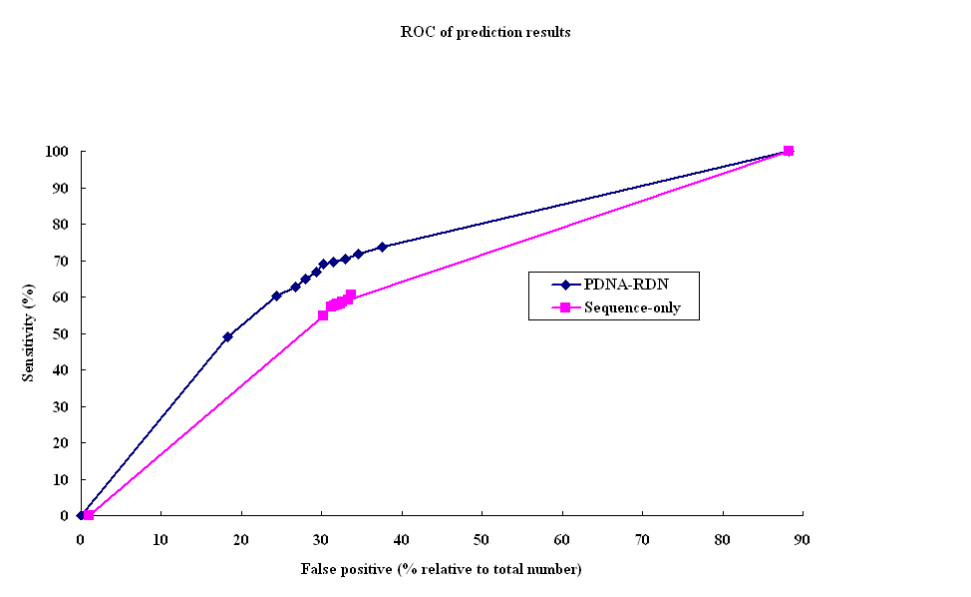
ROC analysis of binding site prediction using PSSMs against PDNA-RDN reference data set, compared with results obtained from sequence based predictions. The sensitivity of the prediction could be adjusted by changing the threshold on predicted probabilities, to annotate that residue to be DNA-binding or otherwise. As may be noted the area under the PSSM based prediction curve is significantly greater than that obtained from sequence based predictions. In addition, sensitivity versus specificity values also seems to be difficult to manipulate in case of sequence based predictions as points on the curve are very closely spaced. PDNA-RDN curve also shows the levels of prediction scores expected on our web-based predictions.

Conservation scores in 20 amino acid positions for every residues form 20 columns (column 3 onwards) of corresponding row in a PSI-BLAST PSSM. For every residue, we make a binary or real-value (interpreted as probability) prediction of that residue being a binding site or not. Input for every prediction is the PSSM score on the row corresponding to this target residue and two more rows on either side, totaling 20 × 5 = 100 inputs (Figure 1).

#### Network architecture and transfer function

We use a neural network with one hidden layer (two nodes) in addition to the input layer described above and a single node output layer. Large number of units in the hidden layer and additional layers were not tried because the data size does not justify an unreasonably large neural network. Network signal is transferred to subsequent layers by an algebraic summation of inputs from the previous layer. Total signal in the last unit is transformed to a real output by a binary decision function much in the same way as in our previous work except that the input to the network is now replaced by PSSM scores rather than 20 bit binary coding [5].

#### Training and validation

A six-fold cross-validation has been used in this work. Out of 62 proteins, 10 were removed at one time and the remaining 52 were trained until the accuracy on the left-out 10 also improved. Six random sets are created in this way and the figures in Table 1 report the averages on all six runs of each set of 10 proteins.

#### Training error function and measure of prediction quality

Data imbalance in the two binary categories for this neural network makes the choice of error function particularly important. We have used an accuracy score called Net Prediction, which is the average of sensitivity and specificity values defined below. Neural network learns to maximize this accuracy score rather than minimizing an error function.

Sensitivity is defined as the number of correct prediction in the binding category relative to total number of such items in the original data and specificity is the number of correctly rejected residues in this category relative to the total number of non-binding residues in the original data.

Sensitivity (S1) = 100 * TP/(TP+FN)     (1)

Specificity (S2) = 100 * TN/(TN+FP)     (2)

where TP: True Positive; TN: True Negative; FP: False Positive; FN: False Negative

Relative number of true positive (TP) values in the prediction was termed as accuracy in our previous work. We have avoided using that term here, as we prefer a more operational definition of accuracy measures here. The imbalance of sensitivity (S1) and specificity (S2) is taken care of by comparing the Net Prediction of the models which gives a better comparison when S1 and S2 vary from one sample to the other. Thus,

Net Prediction (NP) = (S1+S2)/2     (3)

## List of abbreviations

PSSM: Position Specific Substitution Matrix

PSI BLAST: Position Specific Iterative Basic Local Alignment Search Tool

## Authors' contributions

SA conceived and implemented this project. AS contributed in manuscript preparation, results analysis and discussions.

## Figures and Tables

**Table 1:** Prediction results for binding sites in 62 Proteins with different data sets used for generating PSSM.

Reference Data	Overall Correct predictions (%)	Sensitivity (S1) %	Specificity (S2) %	Net Prediction (S1+S2)/2 %
Sequence only (No PSSM)	73.6	40.6	76.2	58.4(2.5)
PDNA-NR90 375 sequences	63.8	65.9	63.4	64.6(2.1)
PDNA-RDN 1386 sequences	64.0	67.1	63.3	65.2(2.1)
NCBI-NR 1,547,365 sequences	66.7	69.5	63.9	66.7(1.4)
PDB-ALL 47,179 sequences	62.6	65.6	61.8	64.7(1.8)
PIR 283,177 sequences	66.4	68.2	66.0	67.1(2.7)

PDNA refers to sequences from Protein-DNA complexes in the Protein Data Bank; NR90 means non-redundant at 90% sequence identity; RDN means data is redundant because similar proteins have not been removed. Values in the brackets show the standard deviation in values obtained from six cross-validation sets. Note that the sensitivity and specificity values shown in this table only refer to those values which sum up to give the best net prediction. These two scores can be mutually adjusted by changing cutoff threshold as described in the text and hence comparison between the data sets should only be made for the net prediction value (the last column) which is the score optimized during training.
